# Apoptotic-like PCD inducing HRC gene when silenced enhances multiple disease resistance in plants

**DOI:** 10.1038/s41598-022-24831-0

**Published:** 2022-11-27

**Authors:** A. C. Kushalappa, N. G. Hegde, R. Gunnaiah, A. Sathe, K. N. Yogendra, L. Ajjamada

**Affiliations:** 1 Plant Science Department, McGill University, Ste. Anne de Bellevue, Quebec, H9X3V9 Canada; 2 International Crops Research Institute for the Semi-Arid Tropics, Hyderabad, Telangana India; 3 Division of Hematology-OncologyJewish General Hospital, McGill University, Montreal, QC Canada; 4 Present Address: University of Horticultural Sciences, Bagalkot, Karnataka India

**Keywords:** Plant stress responses, Biotic, Biotechnology, Plant sciences, Environmental sciences, Oncology

## Abstract

Programmed cell death (PCD) plays an important role in plant environmental stress and has the potential to be manipulated to enhance disease resistance. Plants have innate immunity and, following pathogen perception, the host induces a Hypersensitive Response PCD (HR-PCD), leading to pattern (PTI) or effector triggered immunity (ETI). Here we report a non-HR type or Apoptotic-Like PCD (AL-PCD) in pathogen infected wheat and potato based on apoptotic-like DNA fragmentation. A deletion mutation in the gene encoding histidine rich calcium binding protein (*TaHRC*) in FHB-resistant wheat (R-NIL) failed to induce AL-PCD. Similarly, the CRISPR-Cas9 based silencing of *StHRC* gene in Russet Burbank potato failed to induce apoptotic-like DNA fragmentation, proved based on DNA laddering and TUNEL assays. The absence of AL-PCD in wheat R-NIL reduced pathogen biomass and mycotoxins, increasing the accumulation of resistance metabolites and FHB-resistance, and in potato it enhanced resistance to multiple pathogens. In addition, the reduced expressions of metacaspase (*StMC7*) and Ca^2+^ dependent endonuclease 2 (*StCaN2*) genes in potato with *Sthrc* indicated an involvement of a hierarchy of genes in the induction of AL-PCD. The *HRC* in commercial varieties of different crops, if functional, can be silenced by genome editing possibly to enhance resistance to multiple pathogens.

## Introduction

Unlike animals which have both innate and adoptive immunities, plants rely solely on innate immunity to regulate immune response and cellular suicide called Programmed Cell Death (PCD). PCD is involved in developmental processes and in environmental stresses. Although PCD in animals and plants share several similarities^1,2^ the distinction of PCD types is still challenging, especially in plants, as the molecular mechanisms and the hierarchy of genes involved in driving each type remain elusive^3^. In animals, several types of PCDs have been identified, including apoptotic forms and non-apoptotic forms (*e.g.* autophagy, necroptosis, pyroptosis and ferroptosis)^4^. In plants, following perception of pathogen/microbe/damage associated molecular patterns (PAMPs/MAMPs/DAMPs/toxins) and effectors, the host induces Hypersensitive Response type of PCD (HR-PCD), leading to pattern (PTI) and effector triggered immunity (ETI), respectively^5^. The HR is a dead brown cell with visible lesion in response to pathogen invasion, and the recognition of pathogen by plant leads to triggering of several downstream genes, reinforcing their cell walls to induce HR-PCD. The failure to induce PTI and ETI lead to susceptibility. However, these distinctions are not that clear and the invading pathogen in host encounters several stepwise interventions in host leading to a degree of host susceptibility or resistance^6,7^. DNA laddering is the hallmark of HR-PCD^3,4^. Classic apoptosis, pyroptosis and necroptosis are not observed in plants as in animals but an apoptotic-like PCD (AL-PCD) has been observed. Both the HR-PCD and AL-PCD in plants involve cytoplasmic shrinkage, chromatin condensation, DNA fragmentation, mitochondrial swelling, vacuolization, and chloroplast disruption^8^. However, in HR-PCD the plasma membrane remains intact, whereas in AL-PCD there is plasma membrane blebbing and a characteristic DNA break up^1,3,9,10^. The DNA fragmentation can be a simple DNA cleavage or cleavage into large 50 kb fragments and/or multimer fragments of 180–200 bp. dsDNase, ssDNase and RNase have been reported to induce PCD in potato infected with *Candidatus liberibactor* causing zebra chip^11^. NUCIII nucleases were associated with HR-PCD in tobacco infected with TMV^12^. The endonuclease, *AtCaN2* plays a negative role against abiotic stress in Arabidopsis^13^. The membrane blebbing and breakdown has been attributed mainly due to cysteine proteases, called metacaspases. Based on structural differences, the plant metacaspases have been grouped into two types: type-I containing P20 (20 kDa) and type-II containing P10 (10 kDa) caspase-like subunits; the metacaspases have both cytosolic and nuclear localization signalling domains^14,15^. In Arabidopsis, the AtMC1-3 belongs to type I and the AtMC4-9 belongs to type-II, whereas in *Solanum tuberosum StMC1-6* belong to type-I and *StMC7,8* belong to type-II^15,16^. Several metacaspases were either positively or negatively responsive to biotic stresses^15^. In Suwon11 wheat the *TaMC4* increased PCD, thus the resistance, and when silenced the PCD reduced, leading to increased susceptibility to *P. striiformis* f. sp. *tritici*^17^.

Like in animals, the apoptotic-like pathways in plant cells involve extrinsic or intrinsic stimuli^18^. The extrinsic pathway involves membrane immune receptor *R* gene mediated stimuli, while the intrinsic pathway involves a diverse array of non-receptor-mediated stimuli, including radiation, toxins, viral infections, hypoxia, hyperthermia, and free radicals, and involves intracellular sub-compartments such as mitochondria, nucleus, or others^4^. The PCD limits food supply to biotrophs, whereas hemibiotrophs and necrotrophs feed on dyeing or dead cells, spread further and produce more toxins, some of which can inhibit the synthesis of resistance biochemicals, leading to higher disease severity^19,20^.

Calcium ion (Ca^2+^) is the most prominent second messenger in eukaryotes. Under environmental stress, both abiotic and biotic, calcium ions are generated in the apoplast, and the Ca^2+^ permeable cation channels regulate influx of Ca^2+^ from the apoplast into the cytosol, where the stimulus-responsive Ca^2+^ binds to calcium sensor proteins^21^. Plants possess a few major groups of Ca^2+^ sensors, including calmodulin (CaM) CaM-like proteins (CMLs), Ca^2+^-dependent protein kinases (CDPKs) and calcineurin B-like proteins (CBLs)^22–24^. The Ca^2+^ binding domains consist of helix-loop-helix motif (EF-hands) to which a single Ca^2+^ ion can bind^22,25,26^. When the Ca^2+^ concentration reaches a toxic level, it binds to ATPases (Ca^2+^-ATPases) and transported to different organelles to maintain Ca^2+^ balance^21,22^. The Ca^2+^-CaM, triggers conformational changes, binds to several downstream proteins (Calmodulin binding proteins, CaMBP) at the N-terminal Spatial Ca^2+^ Transforming Element (xWxxx(I or L)xxxx, NSCaTE), and negatively or positively regulates biotic stress resistance^22,27^.

The Sarcoplasmic/endoplasmic histidine rich Ca^2+^ binding protein (HCP or HRC) was first reported in rabbit and then in other animals^28^, and it possesses an acidic surface and a carboxyl-terminal region to which Ca^2+^ binds^29^. *TaHRC* has a NSCaTE binding site where the Ca^2+^ responsive CaM can bind, and hence it is also a Calmodulin binding protein^27^. Plants and/or pathogens vary in their requirements in Ca^2+^ concentration in cytosol for metacaspase activity^14^. The intensity or duration of caspase signaling are critical in determining the death or non-death cell proliferation, where a low intensity leads to PCD and a high intensity leads to cell proliferation^30^. In animals, the *HRC* is an oncogene and leads to cell proliferation and/or apoptosis, depending on the downstream genes activated and the site of Ca^2+^ accumulation^31,32^. The pathways to induce PCD in animals is complex and involves a network of interconnected modules whose targeting may have therapeutic beneficial effects on cancer^33^. Cell proliferation and gall formation is also common in many plant species, following biotic stress^34^.

In plants, the *HRC* gene was first reported to be located within the quantitative trait loci (QTL) *Fhb1* region of wheat near isogenic lines (NILs)^35^. The functional *TaHRC* gene in fusarium head blight (FHB) susceptible near isogenic lines (S-NIL) induced PCD but not in the FHB-resistant line (R-NIL) with the mutated *Tahrc* gene, originating from the Sumai-3 cultivar. Later, this gene was sequenced and deletions in both coding and promoter regions were identified^36^. The *TaHRC* was functional in several wheat genotypes but was naturally mutated in a few land races. This gene was considered a susceptibility gene because the plants with *TaHRC* were susceptible whereas those with mutated *Tahrc* alleles were FHB resistant. Further, *TaHRC* was reported to have a nuclear localization signal^37^. However, another study reported that the mutation within *TaHRC* gene was responsible for FHB resistance in wheat, thus considered this to be a resistance gene^38^. HRC protein was also characterised in *Leymus chinensis* (*LcHRC*) which modulates abscisic acid (ABA)-responsive gene expression through an interaction with histone deacetylation protein (*AtPWWP3*)^39^. They reported that the LcHRC possessed binding sites for both Ca^2+^ and Zn^2+^.

Here we report the induction of AL-PCD in wheat and potato genotypes possessing functional *HRC* gene but not when this gene was mutated (*hrc*). The plants with *StHRC* induced DNA laddering and/or TUNEL positive response but not in plants with *Sthrc*. The expression of metacaspase gene (*StMC7*) was significantly higher in potato with *StHRC* relative to *Sthrc*, when inoculated with both *P. infestans* and *A. solani*. Similarly, the expression of Ca^2+^ responsive endonuclease gene (*StCaN2*) was significantly higher in potato with *StHRC* relative to *Sthrc*, when inoculated with *P. infestans* and *A. solani*. Plants inoculated with different pathogens showed high fold reduction in disease severity and/or pathogen biomass when the *HRC* was mutated, thus demonstrating that the silencing of *HRC* can lead to multiple disease resistance.

## Results

### The wheat NILs with QTL-Fhb1 varied in susceptibility to FHB

**Figure 1:**
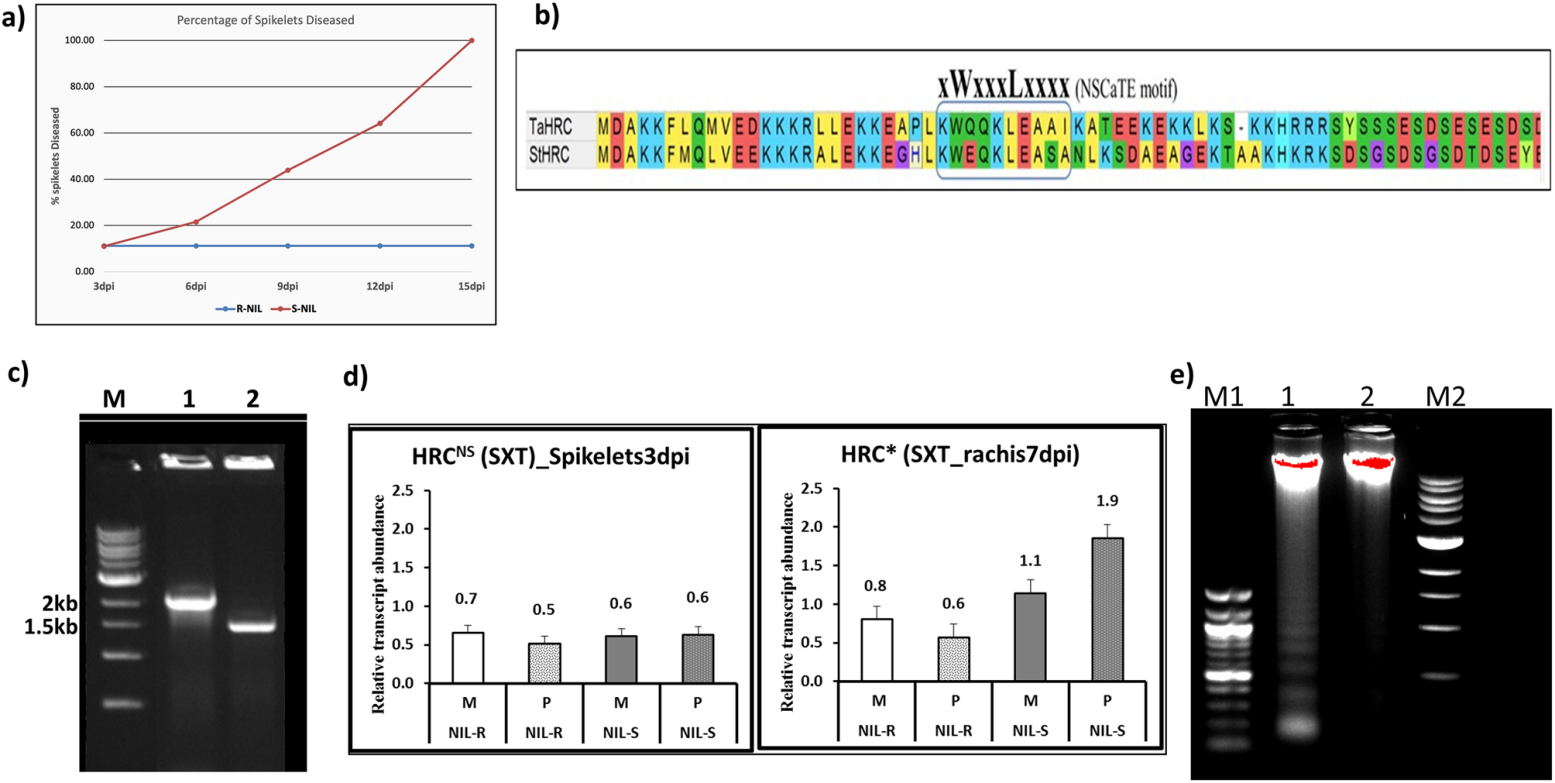
Wheat NILs possessing functional and mutated histidine rich Ca^2+^ binding protein (*TaHRC* or *Tahrc*), inoculated with *F. graminearum*. (**a**) FHB disease progress, based on percent of spikelets diseased, in wheat resistant and susceptible NILs inoculated with *F. graminearum.* (**b**) *TaHRC* (Chinese spring wheat) and *StHRC* (Russet Burbank potato) genome sequences with CaM binding site, NSCaTE. (**c**) The DNA size of the wheat genes *TaHRC* in S-NIL (1) and *Tahrc* in R-NIL (2), M = 1 kb DNA marker ladder; (**d**) *TaHRC* and *Tahrc* gene expression in wheat FHB susceptible S-NIL and resistant R-NIL inoculated with *F. graminearum,* where M is mock, P is pathogen inoculated; (**e**) DNA laddering in wheat NILs inoculated with *F. graminearum*, the original gel pictures, M = DNA ladder, 100 base pairs (M1) and 1 kb (M2), 1 = *HRC* in S-NIL and 2 = *hrc* in R-NIL of wheat.

Wheat near-isogenic lines (NILs) possessing Fusarium head blight (FHB) resistance and susceptible alleles at QTL-Fhb1 region, with FHB-resistance background, were inoculated with *F. graminearum* and the FHB disease severity was assessed. In the FHB resistant wheat NIL, the pathogen failed to spread from the inoculated pair of spikelets to the other spikelets in a spike through the rachis (AUDPC = 1.5; high type II resistance), whereas in the FHB susceptible NIL, the pathogen spread to all spikelets in the spike (AUDPC = 5.6), with 3.7 fold change in resistance (Fig. 1a).

### Identification of a *HRC* gene in wheat and DGEs

The exploration of wheat genome sequence, in the QTL-Fhb1 region, identified the sarcoplasmic/endoplasmic histidine rich Ca^2+^ binding protein (*TaHRC*) as a candidate gene, and it was designated as calmodulin binding protein (*TaCaMBP;* gene ID: *TAA_ctg0954b.00390.1* (Supplementary Fig. S1) (Current: TraesCS3B02G019900) because the *TaHRC* in the Chinese spring genome had a Ca^2+^-CaM binding site, NSCaTE (Fig. 1b). A phylogenetic analysis of *HRC* genes classified wheat *HRC* in Clade-IV (Fig. S1b). The amplified gene length *TaHRC* was 2 kb in FHB susceptible NIL but was truncated in resistant NIL (1.5 kb), indicating a mutation in the latter NIL (Fig. 1c). The gene expression was higher in S-NIL (*TaHRC*) than in R-NIL (*Tahrc*), following *F. graminearum* inoculation (Fig. 1d).

### Characterization of *HRC* in Russet Burbank potato and DGEs

**Figure 2:**
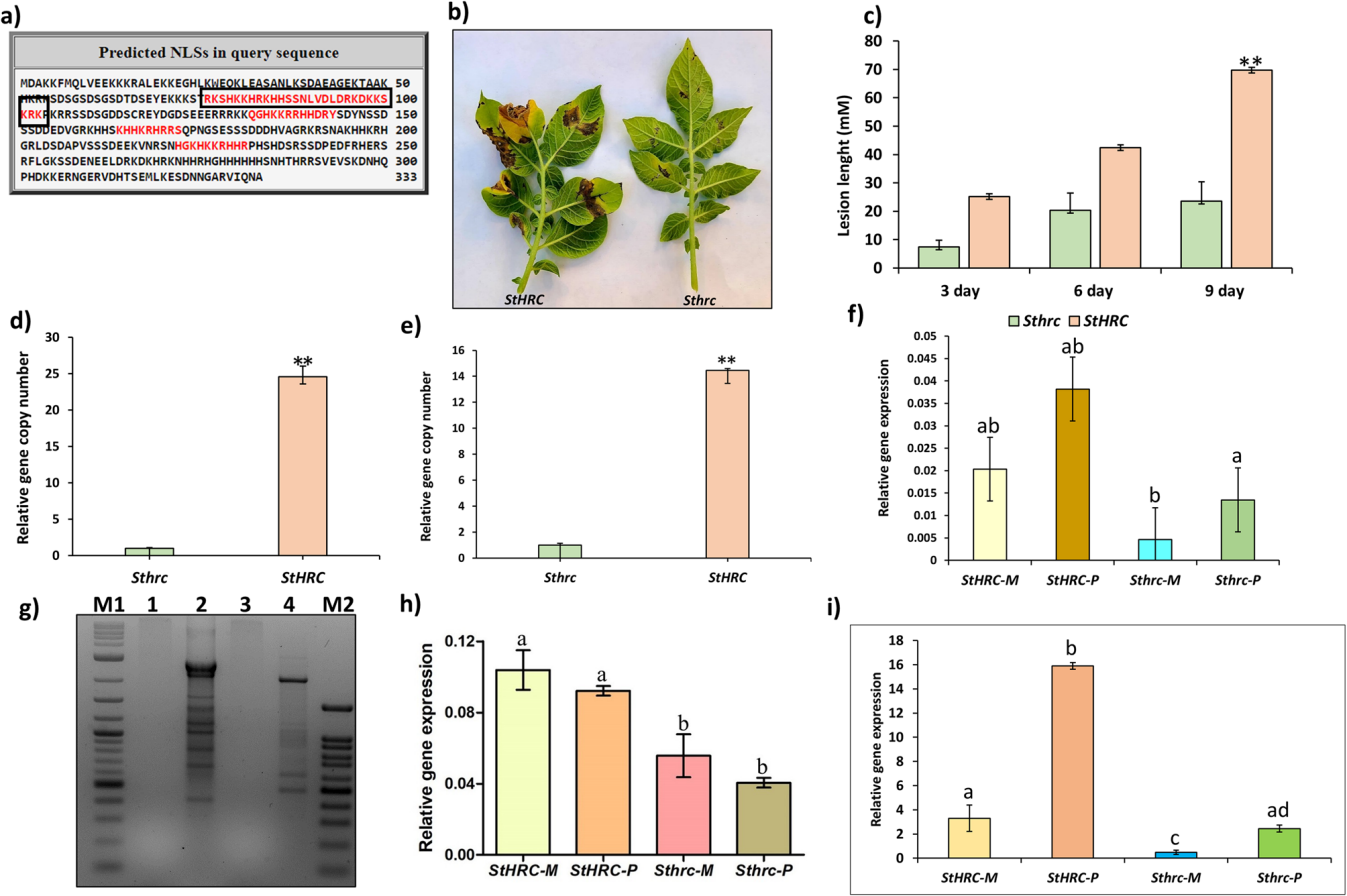
Russet Burbank potato plants, possessing functional and mutated histidine rich Ca^2+^ binding protein (*StHRC* or *Sthrc*), inoculated with *P. infestans*. (**a**) *StHRC* gene sequence with CaM protein binding site, NSCaTE. (**b**) late blight disease symptoms in RB potato with *StHRC* and *Sthrc*; (**c**) disease progress on stem, based on lesion diameter, in RB potato with *StHRC* and *Sthrc*, inoculated with *P. infestans.* (**d**) pathogen biomass in the diseased area of stem; (**e**) pathogen biomass in the diseased area of leaf; (**f**) relative gene expressions of *StHRC* and *Sthrc*, in RB, inoculated with *P. infestans;* (**g**) DNA laddering in RB potato inoculated with *P. infestans*, M is DNA ladder, M1 is 100 bps and M2 is 1 kb, 1 = *StHRC* Mock, 2 = *StHRC* Pathogen, 3 = *Sthrc* Mock, 4 is *Sthrc* pathogen inoculated*;* (**h**) Gene expression of *StCAN2* in RB plants, with *StHRC* and *Sthrc*, following inoculation with *P. infestans;* (**i**) Gene expression of *StMC7* in RB plants, with *StHRC* and *Sthrc*, following inoculation with *P. infestans*. Significance calculated based on Student’s t-test P value: **p < 0.01, *p < 0.05; or based on multiple comparison tests. Values are average of three replicates.

In Russet Burbank (RB) potato the *StHRC* (gene ID: PGSC0003DMG400030513) was sequenced and confirmed it was functional (Fig. S1c). A phylogenetic analysis of *HRC* gene in plants revealed four distinct clades: Clade-I: potato and tomato; Clade-II: tobacco; Clade-III: mainly legumes; Clade-IV: mainly cereals (Fig. S1b). The *StHRC* sequence had both a CaM binding motif, NSCaTE (Fig. 1b) and a nuclear localization signal (Fig. 2a).

### CRISPR-Cas9 based silencing of *StHRC* in RB potato showed multiple disease resistance

**Figure 3:**
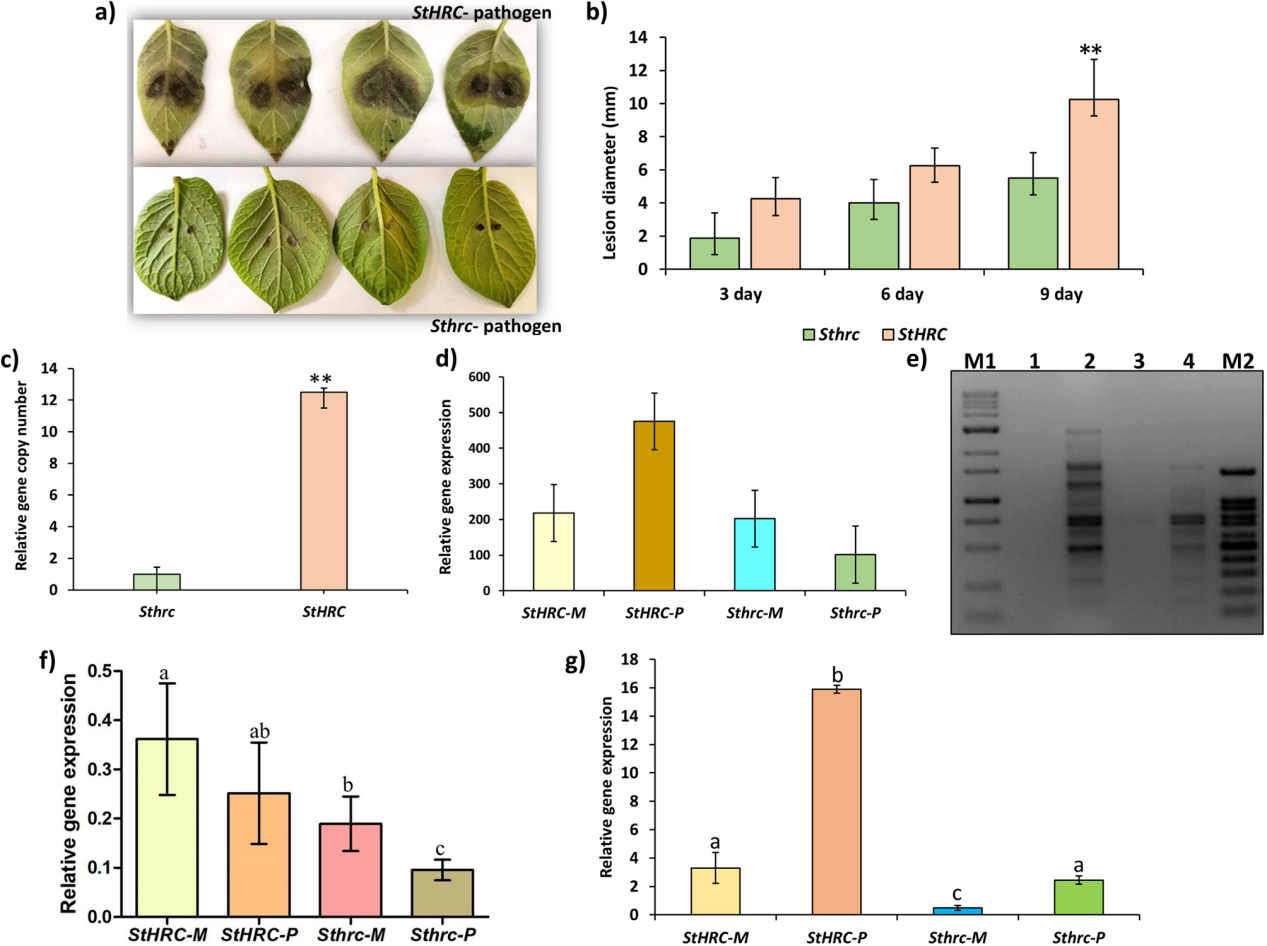
Russet Burbank potato plants, possessing functional and mutated histidine rich Ca^2+^ binding protein (*StHRC* or *Sthrc*), inoculated with *A. solani.* (**a**) Early blight disease symptoms in RB potato with *StHRC* and *Sthrc*; (**b**) disease progress, based on lesion diameter; (**c**) pathogen biomass in the diseased leaf area. (**d**) relative expressions of *StHRC* and *Sthrc* genes in RB potato; (**e**) DNA laddering in RB potato, with *StHRC* or *Sthrc*, inoculated with *A. solani,* M is DNA ladder, M1 is 100 bps and M2 is 1 kb, 1 = *StHRC* Mock, 2 = *StHRC* Pathogen, 3 = *Sthrc* Mock, 4 is *Sthrc* pathogen inoculated; (**f**) Gene expression of *StCAN2* in RB plants, with *StHRC* and *Sthrc*, following inoculation with *A. solani*; (**g**) Gene expression of *StMC7* in RB plants, with *StHRC* and *Sthrc*, following inoculation with *A. solani.* Significance calculated based on Student’s t-test P value: **p < 0.01, *p < 0.05, or based on multiple comparison tests. Values are average of three replicates.

**Figure 4:**
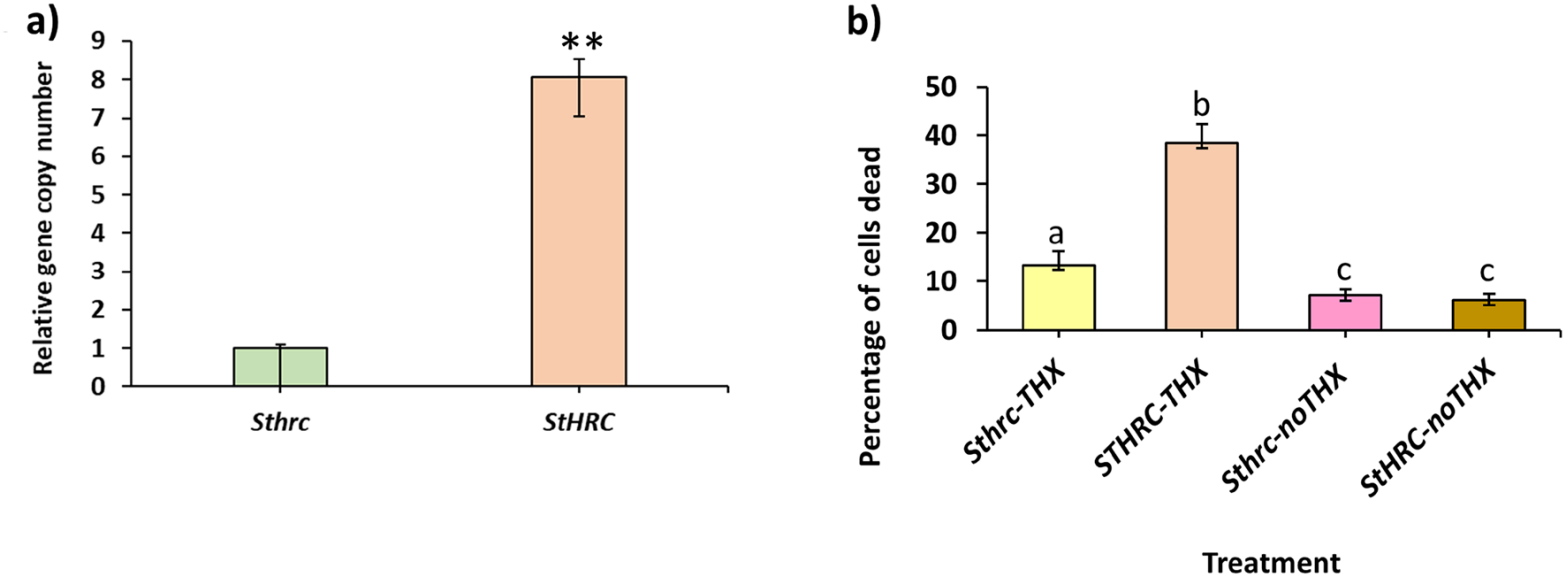
Russet Burbank potato plants or their protoplasts, possessing functional and mutated histidine rich Ca^2+^ binding protein (*StHRC* or *Sthrc*), inoculated with *S. scabiei* or Thaxtomin-A. (**a**) RB potato with *StHRC* and *Sthrc* inoculated with *S. scabiei*: pathogen biomass. (**b**) TUNEL assay of RB potato protoplasts, with *StHRC* and *Sthrc*, inoculated with Thaxtomin-A toxin, produced by *S. scabiei*. Significance calculated based on Student’s t-test P value: **p < 0.01, *p < 0.05 or based on multiple comparison tests. Values are average of three replicates for pathogen biomass quantification and five replicates for TUNEL assay.

To prove the role in disease resistance, the *StHRC* gene in RB potato was silenced based on CRISPR-Cas9 gene editing. The silenced *Sthrc* was Sanger sequenced and silencing of both the alleles of the autotetraploid RB were confirmed (Fig. S1d). The RB potato plants with *HRC* and *hrc* genes were inoculated with mock and *Phytophthora infestans*. At 9dpi the late blight expanded to a large area in RB with *StHRC* but limited to a speck in RB with *Sthrc* gene (Fig. 2b). The disease severity was measured as lesion diameter at 9 dpi, and the fold changes in disease severity was reduced by FCDS = 3.0 in stem of RB with *Sthrc* relative to *StHRC* (Fig. 2c) and the fold changes in pathogen biomass in the diseased area, of RB with *Sthrc* relative to RB with *StHRC*, was reduced by FCBM = 24.6 and FCBM = 14.4, in stem and leaf, respectively (Fig. 2d,e). The differential gene expression (DGE) was significantly higher in RB with *Sthrc* but not with *StHRC*, following pathogen inoculation (Fig. 2f). Similarly, on the leaves of RB with *StHRC* inoculated with *Alternaria solani* developed large lesion at 9dpi but it remained as a speck on the RB with *Sthrc* (Fig. 3a). The disease severity on RB with *Sthrc* relative to *StHRC* was reduced by FCDS = 2.3 and the pathogen biomass was reduced by FCBM = 12.5 (Fig. 3b,c). The DGE was not significant in RB with both *StHRC* and *Sthrc*, inoculated with mock or *A. solani* (Fig. 3d). In tubers of RB inoculated with *Streptomyces scabiei* the pathogen biomass was significantly reduced in *Sthrc* relative to *StHRC* by FCBM = 8.1 (Fig. 4a). Based on these findings we propose that the silencing of *HRC* in plants can confer multiple disease resistance.

### Plants with functional *HRC* but not with the mutated (*hrc*) induced AL-PCD

To prove that the functional *HRC* can induce AL-PCD, the DNA collected from spikelets of wheat NILs possessing functional and mutated alleles inoculated with *F. graminearum* were subjected to DNA laddering, which revealed distinct ladders in FHB susceptible wheat NIL possessing *TaHRC* but not in FHB resistant NILs possessing naturally mutated *Tahrc* (Fig. 1e). Similarly, the RB variety of potato plants with functional (*StHRC*) and silenced (*Sthrc*), based on CRISPR-Cas9 gene editing, inoculated with *P. infestans* and *A. solani* revealed DNA ladders only in plants possessing *StHRC* but not in silenced *Sthrc* (Figs. 2g; 3e). The inoculation of Thaxtomin A, a cellulose biosynthesis inhibiting toxin produced by *S. scabiei*, to protoplasts of RB potato plants with the functional *StHRC* induced AL-PCD, proved based on a terminal deoxynucleotidyl transferase dUTP nick-end labelling (TUNEL) assay, but the absence of Thaxtomin A failed to induce AL-PCD in protoplasts (Fig. 4b).

### Ca^2+^ responsive metacaspases and endonucleases in RB potato and their role in AL-PCD

The hallmarks of AL-PCD are the plasma membrane breakdown and apoptotic-like DNA fragmentation. The cytoplasm shrinkage and membrane breakdown in plants is induced by metacaspases. The *Solanum tuberosum* metacaspase gene (*StMC7*), revealed a significantly higher gene expression in both *P. infestans* and *A. solani* inoculated RB-plants with *StHRC* relative to *Sthrc*, and also was significant in pathogen inoculated relative to mock (Fig. 2i) and *A. solani* (Fig. 3g). An ortholog of the *AtCaN2* in potato genome, belonged to clade-I among the IV clades was identified in potato, designated as *StCaN2* (Fig. S1e). When inoculated with *P. infestans* the DGE of *StCaN2* was significantly higher in *StHRC* relative to *Sthrc* but not significant between the mock and the pathogen inoculated (Fig. 2h). Whereas when inoculated with *A. solani* the DGE was significant higher in *StHRC* than in *Sthrc*, and also in *Sthrc* pathogen inoculated than in mock, but not in *StHRC* (Fig. 3f).

### Resistance metabolites in wheat with *Tahrc* against *F. graminearum*

Metabolic profiling of wheat NILs, inoculated with mock and *F. graminearum*, identified higher abundances of several resistance metabolites mainly in the phenylpropanoid pathway, in FHB resistant NIL possessing mutated *Tahrc* compared to susceptible NIL with *TaHRC* (Table 1a; Supplementary Table S1). The abundance of a protein synthesis inhibitor toxin (deoxynivalenol, DON) was significantly lower in wheat NIL having *Tahrc* (1.2 ppm) than in NIL having functional *TaHRC* gene (2.32 ppm). However, the abundances of several resistance metabolites were higher in NILs with *Tahrc* than with *TaHRC*. The FHB-resistant NIL with mutated *Tahrc*, due to the lack of AL-PCD, was able to biosynthesize significantly reduced amount of DON relative to NIL with *TaHRC*, which has enabled the R-NIL with *Tahrc* to biosynthesize higher amounts of several resistance metabolites.

**Table 1:** Metabolites putatively identified, in spikelets and rachises of wheat NILs with resistant and susceptible QTL-Fhb1 alleles, at 3 (**a**) and 7 (**b**) days post-inoculation with *F. graminearum,* respectively (details in Supplementary Table S2).

a: Induced resistance (IR) metabolites putatively identified in spikelets at 3 dpi				
Sl no.	Observed mass (Da)^a^	Putative names of metabolites	Fold change^b^	
**A. Phenylpropanpoids: free phenylpropanoids, lignans, phenolic glucosides and flavonoids**				
1	148.0525	*trans-*cinnamic acid	1.24*^c^	
2	164.0473	4-coumaric acid	1.45***	
3	206.0579	*p*-coumaroyldiketide	2.09*	
4	210.0891	Sinapyl-alcohol	4.4*	
5	238.084	Sinapic acid methyl ester	2.99*	
6	250.1346	N-Caffeoylputrescine	1.11*	
7	342.097	Caffeic acid 3-glucoside	1.64*	
8	356.1104	1-O-Feruloyl-β-d-glucose	1.49*	
9	358.1411	(+)-pinoresinol	1.24**	
10	372.1203	(+)-sesamolinol	1.81***	
11	414.1277	(–)-Podophyllotoxin	1.5*	
12	520.1936	(–)-Pinoresinol glucoside	1.53**	
13	540.1623	Cleistanthin A	2.34	
14	540.1658	2″-o-p-Coumaroylaloesin	1.35	
15	550.204	Medioresinol 4′-O-β-d-glucopyranoside	1.39	
16	328.1306	2,3,4,6-Tetramethoxychalocone	1.54*	
17	340.1306	6-Prenylnaringenic	1.3**	
18	402.1128	8-p-Coumaroyl-3,4-dihydro-5,7-dihydroxy-4-phenylcoumarin	1.55*	
19	422.1714	Lupinisoflavone G	1.14*	
20	446.1239	Biochanin A-β-d-glucoside	1.34*	
21	478.2769	3-Geranyl-4-2′,4′,6′-tetrahydroxy-5prenyldihydrochalcone	1.38***	
22	500.13	Epigallocatechin 5,3′,5′-trimethyl ether 3-O-gallate	1.2*	
23	564.1068	Isorhamnetin 3-(6″-malonylglucoside)	2.53	
24	580.2147	(+)-Syringaresinol O-β-d-glucoside	1.75	
25	602.2352	5,4′-Dihydroxy-6-C-prenylflavanone 4′-xylosyl-(1→2)-rhamnoside	1.11	
28	726.2354	Naringenin 7-O-(2″,6″-di-O-alpha-rhamnopyranosyl)-beta-glucopyranoside	1.43	
29	742.2666	Acanthoside D or (–)-Syringaresinol di-beta-d-glucoside	1.29	
30	800.2357	Tricin 7-rutinoside-4′-glucoside	1.56	
31	862.2141	Cyanidin 3-[6-(6-p-hydroxybenzoylglucosyl)-2-xylosylgalactoside]	1.75	
**B. Lipids**				
32	136.0374	Threonate	1.24*	
33	172.1464	Caprate	1.78*	
35	438.261	(–)-Fusicoplagin A	2.74*	
36	440.2766	PA(17:1(9Z)/0:0);1-(9Z-heptadecenoyl)-sn-glycero-3-phosphate	3.11*	
39	600.2312	12S-acetoxy-punaglandin 2	1.56	
**C. Terpenoids and alkaloids**				
40	154.1359	(3S)-linalool	1.29*	
41	240.0898	β-Carboline-1-propionic acid	1.3*	
42	376.1361	Loganate	1.59*	
43	376.1516	Euparotin	1.08***	
44	376.1516	Ailanthone	1.54**	
45	418.1623	Euparotin acetate	1.28**	
46	522.2091	Isobrucein A	1.46*	

b: Glycerophospholipids putatively identified in rachis at 7dpi				
Observed mass (Da)	Putative names of metabolites	RM/SM	RP/RM	SP^d^/SM
432.2265	PA(18:3(6Z,9Z,12Z)/0:0)	1.01	0.94	0
453.2846	PE(16:0/0:0)	1.05	1.04	0
479.3001	PC(15:1(9Z)/0:0)	0.99	0.91	0
503.3	PE(20:3(8Z,11Z,14Z)/0:0)/LysoPE(0:0/20:3(11Z,14Z,17Z))	0.97	1.00	0
505.3154	PC(17:2(9Z,12Z)/0:0) or LysoPE(0:0/20:1(11Z))	1.07	0.93	0
507.3315	PC(17:1(10Z)/0:0)	1.01	0.90	0
508.3132	PG(19:0/0:0): 1-nonadecanoyl-glycero-3-phospho-(1′-sn-glycerol)	0.97	1.0	0
519.2594	PS(18:3(6Z,9Z,12Z)/0:0)	0.97	0.97	0
555.3524	PC(17:0/0:0);1-heptadecanoyl-sn-glycero-3-phosphocholine	0.96	1.12	0
559.2872	PS(19:1(9Z)/0:0): 1-(9Z-nonadecenoyl)-glycero-3-phosphoserine	1.06	0.96	0
567.3523	PS(21:0/0:0)	1.07	0.86	0
578.4295	PA(O-16:0/12:0)	1.06	0.98	0
581.368	PS(22:0/0:0)	1.07	0.89	0
638.414	PG(14:0/12:0)	0.85	1.17	0
662.4139	PG(14:1(9Z)/14:1(9Z))	0.92	1.12	0

^a^The metabolite mass was included only when the observed mass accurate mass error, AME was < 5 ppm, which was calculated as [(Observed mass − Exact mass)/Exact mass] × 10^6^. To the observed mass one 1H was added, where the mass of H = 1.007276, as the mass was detected based on the negative ionization mode, using LC-HRMS.

^b^Fold change in abundances of induced resistance (IR) metabolites in NIL-R, calculated as the abundance: IR = (RP/RM)/(SP/SM), where R is resistant NIL, S is susceptible NIL, M is mock inoculated, and P is pathogen inoculated.

^c^Significance calculated based on student t-test *P* value: *** < 0.001, ** < 0.01, * < 0.05, NS is Not-significant.

^d^In (b), SP/SM = 0, because these metabolites were absent in the SP, at 7dpi.

### Glycerophospholipids indicate the plasma membrane breakdown in plants with *HRC* infected with *F. graminearum*

The glycerophospolipids are the major constituents of plant cell membrane. Interestingly, the metabolic profiling of wheat NILs inoculated with *F. graminearum* and mock, at 7dpi, revealed several glycerophospholipids, including phosphotidylserines (PS-externalization), in pathogen inoculated resistant NIL having *Tahrc* which were not detectable in pathogen inoculated susceptible NILs having *TaHRC*, depicting plasma membrane breakdown, another hallmark of AL-PCD (Table 1b; Supplementary Table S2).

## Discussion

Trophism in plant pathogens is generally classified into biotrophs that obtain food from living cells, necrotrophs that first kill cells and then obtain food from the dying or dead cells, and the hemi-biotrophs that switch from an initial biotrophic phase to a necrotrophic phase^20^. It is possible that several necrotrophs also may have a short biotrophic phase. The PAMPs and effectors produced by these pathogens are perceived by the host membrane localized pattern recognition receptors (PRRs), that trigger the downstream *R* genes to induce HR-PCD, leading to PTI or ETI^5,7^. Non-HR types of PCD symptoms have been observed in several plan-pathogen interactions, especially in plants infected with necrotrophic pathogens^20^.

The hallmarks of AL-PCD are the breakdown of DNA and membranes, however, the hierarchy of genes involved are still elusive. The Ca^2+^ are generally induced in the apoplast, following infection by biotrophs, hemibiotrophs and necrotrophs^9^. The induced Ca^2+^ in plant acts as signal molecules and regulate downstream resistance genes. Silencing of *HRC* gene in both wheat and potato, significantly reduced the disease severity and the pathogen biomass in the diseased area, following infection by both necrotrophic and hemibiotrophic pathogens. The *HRC* gene, triggered by the CaM, significantly enhanced Ca^2+^ concentration in the cytosol. The increased Ca^2+^ can regulate downstream *R* genes, such as metacaspases and endonucleases, to induce AL-PCD based on two mechanisms: *(1) Membrane blebbing and breakdown:* The plants have metacaspases, cysteine-dependent multifunctional proteins, that are Ca^2+^ responsive and they can induce cell proliferation and breakdown of membrane and DNA, the hallmarks of AL-PCD^14,40^. In potato, the metacaspase gene (*StMC7*) was significantly induced following *Phytophthora* and *Alternaria* inoculations, but the expression was significantly reduced when *StHRC* was silenced. The *StMC7* expression was not completely reduced because the reduction in the amount of Ca^2+^ following *HRC* silencing was not complete, as the CaM can still enhance the concentration of Ca^2+^ in the cytosol. The silencing of *HRC* in animals also failed to completely suppress tumors and this was considered to be due to caspases that may be still functional^31^. In plants, several pathogens and pests induce galls and cell proliferations and silencing of *HRC* may also reduce them, increasing resistance^34,41^. The ZmMC6-8 associate with a maize QTL are known for resistance to several maize diseases. Baculovirus P35 gene that inhibits metacaspases reduced PCD, following inoculation of *A. alternata* f. sp. *lycopersici*, reducing AAL toxin^42^. PIRIN2 a cupin family protein, which can bind to several metacaspases, when silenced, significantly reduced *Ralstonia solanacearum* in Arabidopsis^43^. *(2) DNA breakdown*: The increased Ca^2+^ in cytosol, when reaches a toxic level is transported to other organelles, including nucleus, by ATPases^12,13^. In addition, the *HRC* can also move to nucleus, as it has nuclear localization domain, and can significantly enhance the Ca^2+^ concentration. Plants have several Ca^2+^ responsive endonucleases^13^. In potato also several endonucleases have been reported^3,31^, of which the expression of *StCaN2* was significantly reduced when the *HRC* was silenced, but the reduction was not significant, with *Phytophthora* relative to mock inoculations. This may be because the *HRC* is not the only enhancer of Ca^2+^ in the cytosol and nucleus, it can also be increased by the CaM and ATPases^12,13,17^. The model below describes the possible hierarchy of genes involved in plants to induce AL-PCD.

### Proposed Model for AL-PCD in plants and biotic stress resistance

**Figure 5:**
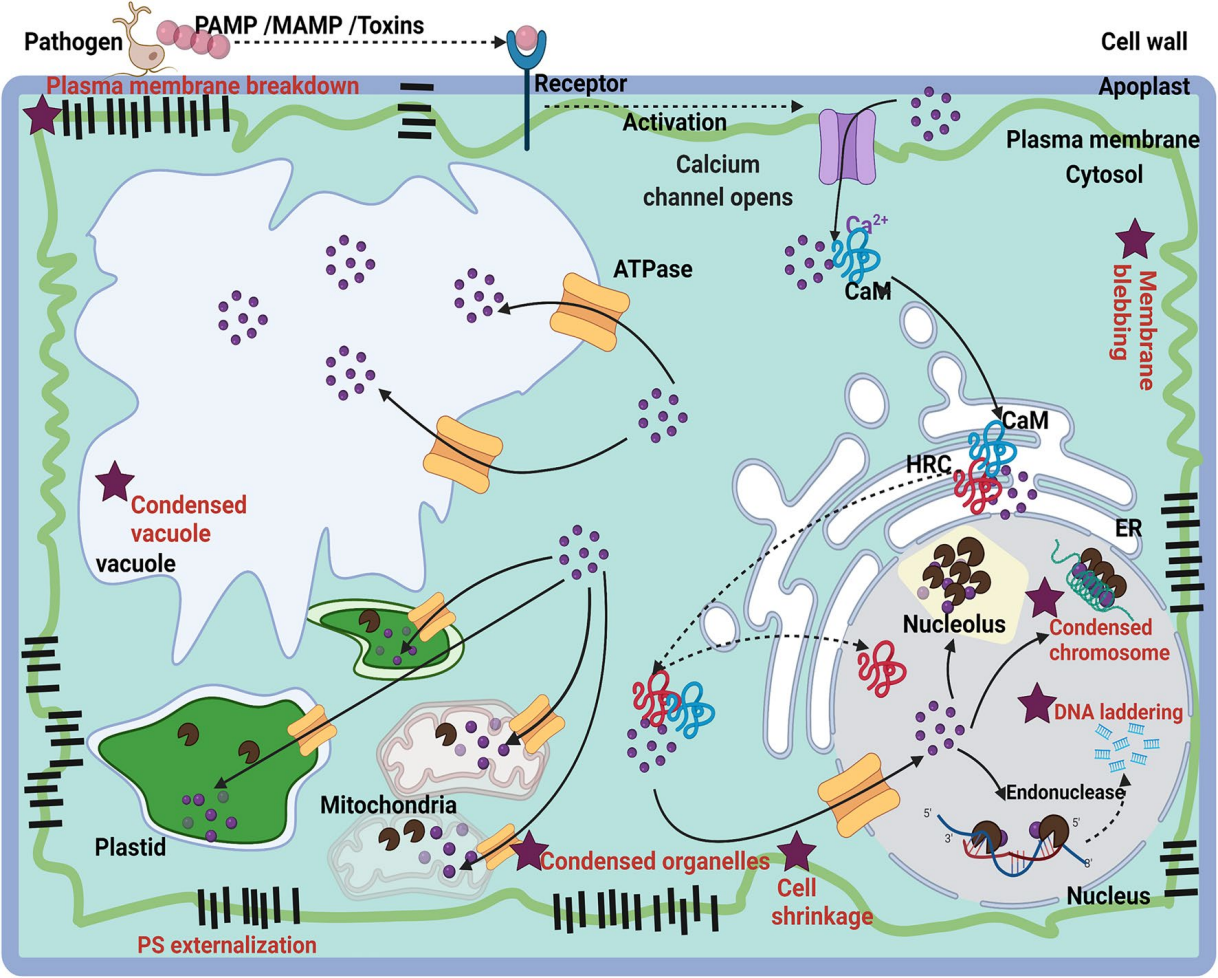
Model for Apoptotic-like PCD (AL-PCD) in plants. The important characteristics of AL-PCD are shown with *: cytoplasm shrinkage, plasma membrane blebbing and breakdown, phosphotidylsterine (PS) externalization, condensed vacuole, plastids and chromosome, and DNA laddering (metacaspases are involved in all thus not shown). Acronyms: Ca^2+^ Calcium ions influx through calcium channels; Ca^2+^ transport by ATPases; CaM, Calmodulin binding protein; ER, endoplasmic reticulum; *HRC,* histidine rich calcium binding protein; PAMP/MAMP, pathogen or microbe associated molecular patterns.

Taken together, we propose that following pathogen invasion, there is an influx of Ca^2+^ from the apoplast into the cytosol^26^, where it binds to calcium sensor proteins such as CaM/CML (CaM-like), possibly to StCaM4 in potato^26^. The Ca^2+^-CaM triggers conformational changes and regulates Ca^2+^ through membrane localized calcium ion channels. In the cytosol, the Ca^2+^-CaM binds at the NSCaTE motive of the HRC protein that is localized at the endoplasmic reticulum (Fig. 5)^19,26,27^. When Ca^2+^ concentration reaches a toxic level, to maintain its balance in cytosol, the cytosolic Ca^2+^ is transported to other organelles by binding to ATPases^21,22^. The increase in Ca^2+^ concentrations in the cytosol and organelles induce the breakdown of plasma membrane and other organelle membranes, resulting in cell shrinkage. The increase in Ca^2+^ concentration in the cytosol by *HRC*, can also trigger Ca^2+^ responsive metacaspases, that can induce breakdown of membranes and DNA^14,40^. The metacaspase, *StMC7*, was highly upregulated in RB with *StHRC*, following inoculation of *P. infestans* and *A. solani*^35^. The *HRC*, having nuclear localization signal (Figs. 1b, 2a), moves to the nucleus and increases Ca^2+^ concentration. In the nucleus, the Ca^2+^ triggered endonuclease, possibly the StCaN2 of the Clade-I, induce AL-PCD (Fig. S1e)^3,13^. In addition, the metacaspases also have nuclear localization signals and they also may induce endonucleases and AL-PCD^14^. The DNA laddering and TUNEL assays depicting apoptotic-like DNA fragmentation, and the disappearance of plasma membrane constituents, such as glycerophospholipids, as observed here in wheat (Table 1b), are the hallmarks of AL-PCD and not HR-PCD^3,4^. Further studies are required to explain the mechanisms how the calcium ion channels, CaM, HRC, ATPases, endonucleases and metacaspases can induce AL-PCD when the *HRC* is functional, and as well, the roles of hierarchies of *R* genes involved in the induction of multiple disease resistance when the *HRC* is silenced (*hrc*)^7^.

Several necrotrophic pathogens produce mycotoxins that supress disease resistance biochemicals biosynthesized by plants^20,44^. The *F. graminearum* produced mycotoxin DON is a protein biosynthesis inhibitor and the mechanism of FHB severity reduction is mainly associated with the amount of DON produced. In wheat susceptible NILs, the dying and dead cells induced by AL-PCD are colonized by the necrotrophic pathogens and increase pathogen biomass increasing the biosynthesis of DON. The increased DON inhibits the protein biosynthesis by the *R* genes, thus disables the plant defence. Whereas in wheat FHB-resistant NILs the *Tahrc* fails to induce AL-PCD and the lack of dying and dead cells for the pathogen to feed reduces the pathogen biomass and thus the amount of DON biosynthesized. Reduced amount of DON decreases the inhibition of protein biosynthesis by the *R* genes, thus facilitating biosynthesis of more resistance biochemicals by the plant *R* gene repertoire of host, thus enhancing the resistance in wheat to FHB. In the R-NIL the mutated *Tahrc* gene significantly increased the accumulation of several resistance metabolites (Table 1A), meaning the biosynthetic *R* genes of these were inhibited by DON produced in S-NIL with *TaHRC* gene that induced AL-PCD. However, the increase in FHB resistance can not be explained only by the mutated *HRC*, as the wheat QTL-Fhb1 localizes 37 genes, of which four genes have been reported to induce FHB-resistance, however, among these only the *TaHRC* can induce AL-PCD, and the other three genes are: *TaPFT*, a pore forming toxin gene, *TaLAC4*, a monolignol biosynthetic gene which are then deposited to reinforce the secondary cell walls to contain the pathogen, and *TaNAC032*, a transcription factor that enhances *TaLAC4* gene^45–47^. Similarly, the *S. scabiei* infects potato tubers and produces a toxin, Thaxtomin-A, which is a cellulose biosynthesis inhibitor, and the lack of Thaxtomin-A accumulation in tubers reduced scab formation^48^. *Alternaria* spp*.* are known to produce Alternaric acid, Zinniol, solanapyrones, etc. which can interfere with host metabolism and the expression of *R* genes^49^. Arachidonic acid, an elicitor fatty acid produced by *P. infestans* in infected potato and tomato, triggered AL-PCD^50^. It is also known to produce a small cysteine-rich (scr74 gene family) phytotoxic protein, similar to PcF (*Phytophthora cactorum* in *Fragaria*) toxin^51^. In plants with functional *HRC* gene, the incorporation of new *R* genes may not show significant increase in resistance against necrotrophic pathogens, unless the *HRC* or metacaspase and/or endonuclease, is silenced to avoid AL-PCD. It would be interesting to see the hierarchies of potato genes involved, following inoculations of specific races of *P. infestans*, in inducing HR-PCD and AL-PCD.

It is possible that at low Ca^2+^ concentration the host induces HR-PCD, following biotrophic infection, whereas at high Ca^2+^ concentration, accumulated by *HRC*, the host induces AL-PCD, in response to necrotrophs^14,30^. The necrotrophs evolved to feed on these dying or dead cells and cause a higher disease severity. In nature, the *HRC* gene in wheat Sumai-3 and in a few other land races, the hosts also evolved, mutated the *HRC* gene and enabled biosynthesis of resistance metabolites and proteins. The silenced *StHRC* gene reduces the Ca^2+^ concentration in both the cytosol and nucleus, reducing the metacaspase and endonuclease activities, which significantly reduces the induction of AL-PCD leading to enhanced multiple disease resistance. However, the *StHRC* silencing failed to completely suppress both the expression of genes, the *StMC7* and *StCaN2*, thus the reduction in AL-PCD was not complete or relatively less. This is because the silenced *StHRC* was unable to completely stop accumulation of Ca^2+^, neither in the cytosol nor in the nucleus, because of the Ca^2+^ transporters, CAM/CAML and ATPase. Accordingly, instead of silencing *HRC*, it is possible that the silencing of specific endonuclease (*StCaN2*) and metacaspase (*StMC7*), could lead to a higher reduction in DNA and membrane breakdown, which might lead to almost complete suppression of the AL-PCD. The silencing of *HRC* or metacaspases and endonucleases in plants might also reduce cell proliferation and gall formation by several plant pathogens and insect pests^24^. When these are confirmed, if these genes are functional in different varieties of crop plants, they can be silenced based on genome editing, to enhance resistance to multiple pathogens, to achieve sustainable crop production^7^.

## Materials and methods

### Plant material

The wheat near isogenic lines (NILs), with resistant (R) and susceptible (S) QTL-Fhb1, were developed from Sumai-3/Thatcher^52^ (seeds provided by Dr. S. Fox, AAFC, Winnipeg). Both R-NIL and S-NIL were homozygous resistant for the other reported type II FHB resistance QTLs on chromosomes 5A (QTL-Fhb5) and 6B (QTL-Fhb2) from Sumai-3. The NIL resistant to fusarium head blight (FHB) had high type II or resistance to spread of pathogen through rachis from the inoculated spikelet to other spikelets in a spike.

The Russet Burbank (RB) potato is a processing variety, and the tissue cultured plantlets were obtained from the tissue culture centre, New Brunswick, Canada. The RB potato is on public domain. Experimental research, including the collection of plant material, complies with relevant institutional, national, and international guidelines and legislation and appropriate permissions were obtained for the collection of plant material.

### *HRC* gene expression, sequence, and phylogenetic tree in wheat and potato

The physical location of the wheat QTL-Fhb1 has been variedly mapped to a 2.2–2.8 Mb region on the contig ctg0954 (Genbank: FN564434)^53^. An in-silico analysis revealed a gene encoding putative Sarcoplasmic/endoplasmic histidine rich Ca^2+^ binding protein at QTL-Fhb1 (Supplementary Table S1).

The total RNA was extracted using a RNeasy Plant mini kit (Quiagen). 500 ng of total RNA from each sample was reverse transcribed to cDNA using iScript cDNA synthesis kit (BioRad, ON, Canada). Two microliters of 40x-diluted cDNA in 10 µl reaction volume was used in a quantitative PCR (q-PCR) reaction using iQ SYBR Green Supermix (BioRad) in an CFX384TM Real-Time System (BioRad, ON, Canada). In wheat, relative transcript abundance in each sample was calculated by plotting onto the standard curve and was normalized with the reference gene, Ta2291 (*T. aestivum* ADP-ribosylation factor). In potato, normalisation was performed with the reference gene, β-tubulin (Stβ-tubulin) and elongation factor-1α (StEF1α)^54^. Relative transcript abundance between treatments was calculated 2^–∆∆CT^ method^55^ and compared by one-way ANOVA using SigmaPlot 12.5.

The *HRC* gene was searched in the potato genome and RNA-seq of pathogen inoculated Russet Burbank^56^, which was later Sanger sequenced in RB potato (Supplementary Table S1). The *StHRC* gene in RB potato was knocked out (*Sthrc*) based on CRISPR-Cas9. Guide RNA for *StHRC* Russet Burbank was designed using CRISPR-P 2.0^57^ and was cloned in pDIRECT21A^58^. The final CRISPR-Cas9 constructs were delivered to potato internodes using *Agrobacterium* and plants were regenerated from internodal callus selected on Hygromycin^59^. The CRISPR induced mutation of all the four alleles of this gene was confirmed, and the CG1 (Clonal generation1) plant with biallelic mutation was selected for plant propagation, tuber production and disease quantification. The non-silenced (*StHRC*) and silenced (*Sthrc*) plants were further multiplied by tissue culture, in magenta boxes, containing MS (Murashige and Skoog) media and used in this study^59^.

The phylogenetic analysis of the reported HRC protein sequences was performed in Geneious prime using the Genius plugin PhyML^60^ and a Kimura 2-parameter substitution model with bootstrap value of 1000.

### Metacaspase in potato and differential gene expression

The *Solanum tuberosum* metacaspase, *StMC7* (Gene ID: Sotub09g030720.1.1; Ortholog in Arabidopsis At1g79340.1) sequence^16^ in RB potato was sequenced and confirmed they were identical. The RB potato plants with *StHRC* and *Sthrc* were inoculated with *P. infestans,* and *A. solani* and the differential gene expressions were determined.

### Endonucleases in potato and differential gene expressions

The endonuclease gene *AtCAN2* sequence^13^ was used to identify the ortholog in potato. A phylogenetic tree and amino acid sequence analysis identified four Clades and the *StCaN2* belonged to the Clade-I. The RB potato plants with *StHRC* and *Sthrc* were inoculated with *P. infestans* and *A. solani* and the differential gene expressions were determined.

### Disease severity and pathogen biomass assessment

A pair of wheat spikelets in the middle region of spikes of both the NILs were inoculated (at 50% anthesis) with *F. graminearum* (GZ3639, FgTri5 + obtained from Dr. Proctor, USDA, USA) macroconidial suspension (10^5^ spores ml^−1^) and the disease severity was quantified as per cent of spikelets in a spike diseased (PSD), at 3-day intervals, over 15 dpi. From the PSD, the area under the disease progress curve (AUDPC) was calculated^35^.

The leaves of RB potato plants, with functional *StHRC* and silenced *Sthrc* genes, were inoculated with *Phytophthora infestans* and *Alternaria solani* (obtained from Dr. A. Dionne, MAPAQ, QC) spore suspensions, and covered with plastic bags for 48 h. The disease lesion diameter was measured at 7 dpi; the cork borer discs containing the diseased area were collected at 7 dpi, and the pathogen biomass was quantified based on qRT-PCR assay with pathogen specific primers^59,61^. Young tubers were inoculated with *Streptomyces scabiei* (isolate EF-35, obtained from Dr. C. Beauleau, Sherbrooke University, QC, Canada). The 1 cm diameter cork borer discs were collected at 7 dpi and the bacterial biomass was determined based on real-time PCR assay using pathogen specific primers^59,62^.

### DNA laddering in wheat NILs and Russet Burbank potato plants, with *HRC* and *hrc* genes, inoculated with pathogens

Three alternative pairs of spikelets of NILs, with *TaHRC* and *Tahrc*, were inoculated with *F. graminiarum*, and the rachis samples were collected at 7dpi. Genomic DNA (gDNA) was extracted from the rachis, following 7 dpi with *F. graminiarum*, of wheat NILs using DNeasy Plant Mini Kit (Qiagen GmbH, Hilden, Germany). 25 µg of DNA from pathogen inoculated NIL-R and NIL-S, 1 µg each of 100 bp and 1 Kb (Invitrogen, Carlsbad, CA, USA) were resolved on 2% agarose gel made with TAE buffer and stained with ethidium bromide. Gel images were captured using Molecular Imager ChemiDoc XRS System (Bio-Rad Laboratories).

The leaves of RB potato plants, with *StHRC* and *Sthrc*, were inoculated with *P. infestans* and *A. solani*. At 7dpi, a 1 cm diameter cork borer was used to cut the diseased areas, ground and the genomic DNA was extracted using DNeasy Plant Mini Kit (Qiagen GmbH, Hilden, Germany). Genomic DNA was used to perform Ligation-Mediated Polymerase Chain Reaction (LMPCR) and DNA laddering^63^.

### TUNEL assay for AL-PCD

Protoplasts isolated from *StHRC* and *sthrc* plants. *StHRC* plants were inoculated with methanol (control) and Thaxtomin A (2uM) (abcam Inc). TUNEL assay was performed after 72 h of Thaxtomin A treatment, using the dead end colorimetric TUNEL system (Promega corporation). Total of 500 cells were counted (in three replications) to calculate percentage of cell death^48^.

### Metabolic profiling to identify and quantify metabolites

Three alternate spikelets of wheat NILs were inoculated with mock or *F. graminearum* spore suspension and the spikelets and rachis from inoculated regions were separately harvested. The inoculated spike area was collected at 3 and 7 dpi, spikelets and rachis were separated, and metabolites were extracted and analyzed using liquid chromatography high resolution mass spectrometry (LC-ESI-LTQ-Orbitrap, Thermo Fisher, Waltham, MA)^64,65^. From the peak area or the abundances of metabolites, the Constitutive Resistance (CR) and Induced Resistance (IR) metabolites were identified^66^.

### Research involving plants

All the relevant institutional, national, and international guidelines and legislation have been followed.

## Supplementary Information

Supplementary Figure S1.

Supplementary Table S1.

Supplementary Table S2.
