# Supplementary material for: Apoptotic-like PCD inducing HRC gene when silenced enhances multiple disease resistance in plants
Source: Sci Rep. 2022 Nov 27;12:20402. doi: 10.1038/s41598-022-24831-0 (PMC9701806; doi:10.1038/s41598-022-24831-0)

Scientific reports

Apoptotic-like PCD inducing HRC gene when silenced enhances multiple disease resistance in plants

Kushalappa AC[1,5*], Hegde NG[1,5], Gunnaiah R[1,2,5], Sathe A[1], Yogendra KN[3], and Ajjamada L[4]

[1]Plant Science Department, McGill University, Ste. Anne de Bellevue, Quebec, Canada H9X3V9

[2]Curent address: University of Horticultural Sciences, Bagalkot, Karnataka, India.

[3]International Crops Research Institute for the Semi-Arid Tropics, Hyderabad, Telangana, India.

[4]Division of Hematology-Oncology, Jewish General Hospital, McGill University, Montreal, QC, Canada

Supplementary information: Fig. S1. a) Wheat QTL-Fhb1 Candidate genes; b) Phylogenetic tree of HRC in selected plant species, showing four clades, where *StHRC* belongs to Clade I and the *TaHRC* belongs to Clade IV; c) Sangar sequence of *StHRC* gene in Russet Burbank potato; d) CRISP-Cas9 based silencing of *StHRC* gene (*Sthrc*), showing deleted alleles; e) Phylogenetic tree of endonuclease gene StCaN2 in selected plant species, showing four Clades, where the *StCAN2* belongs to Clade I; f) Amino acid sequence alignment of *StCAN2* genes in plants. The putative staphylococcal nuclease (SNase) catalytic domain is shown in black box. Black circles indicate Ca2+ binding site. The open circles show the active site of the SNase catalytic domain.

Raw data on metabolites detected is in Supplementary information. Any additional data, with reasonable request, is available from the corresponding author.

a) # *Fhb1* candidate genes

Selected genes localized in the wheat QTL-Fhb1 region.

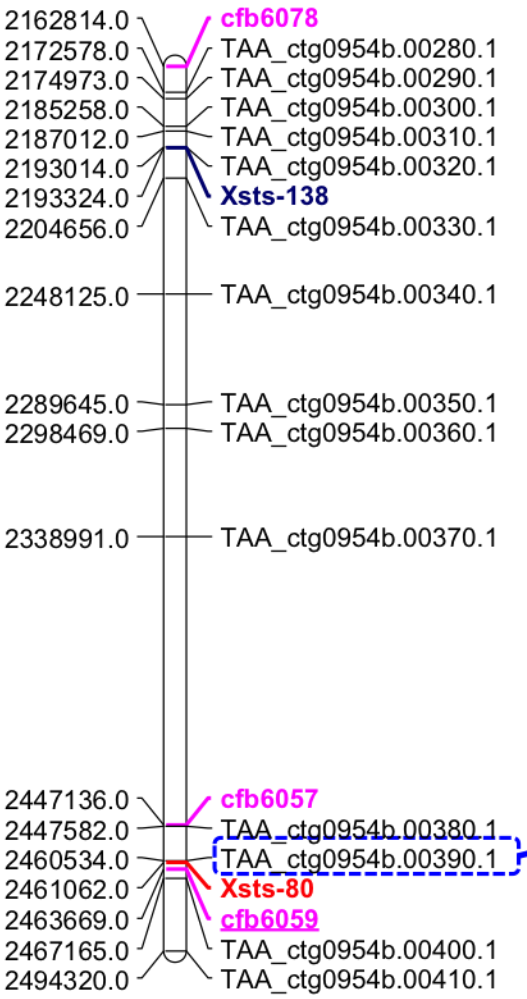

Gene	Protein
TAA_ctg0954b.00280.1	Glycosyltransferase, HGA-like
TAA_ctg0954b.00290.1	Leucyl-tRNA synthetase
TAA_ctg0954b.00300.1	Exonuclease
TAA_ctg0954b.00310.1	Alanyl-tRNA synthetase
TAA_ctg0954b.00320.1	conserved hypothetical protein
TAA_ctg0954b.00330.1	PAP fibrillin domain containing protein
TAA_ctg0954b.00340.1	tRNA (guanine-N(1)-)-methyltransferase
TAA_ctg0954b.00350.1	UDP-glucose 6-dehydrogenase
TAA_ctg0954b.00360.1	Polygalacturonase precursor
TAA_ctg0954b.00370.1	Oxidoreductase NAD-binding domain containing protein
TAA_ctg0954b.00380.1	Terpene synthase
TAA_ctg0954b.00390.1	**Sarcoplasmic reticulum histidine-rich calcium-binding protein precursor (HRC)**
TAA_ctg0954b.00400.1	Conserved hypothetical protein (HCT

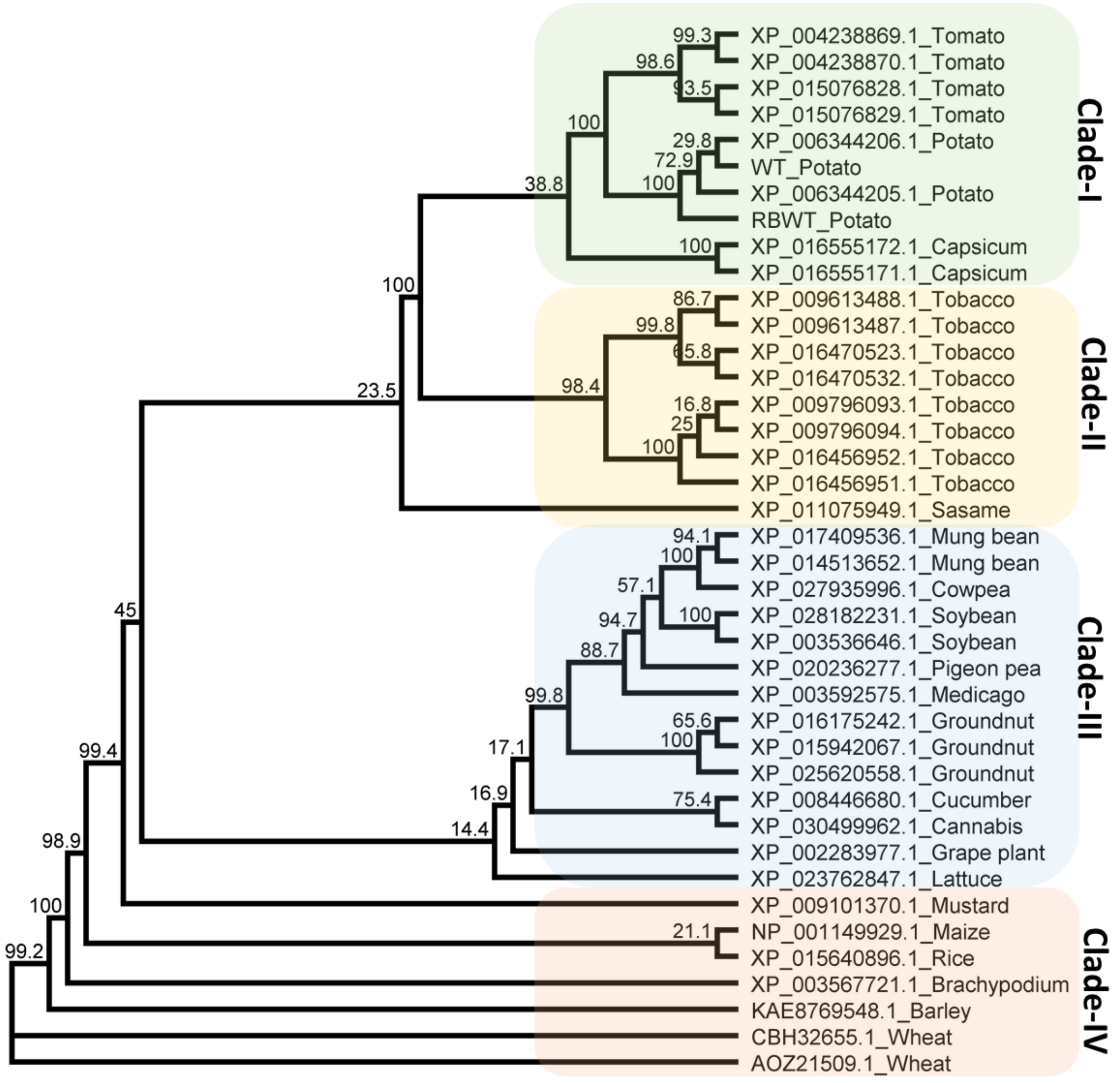

b) Phylogenetic tree of *HRC in selected plant species, showing four clades, where StHRC belongs to Clade-I and the TaHRC belongs to Clade-IV.*

c) Sangar sequence of *StHRC* gene in Russet Burbank potato

```
          1       10        20        30        40        50        60        70        80        90        100       110       120       130
          |--------+---------+---------+---------+---------+---------+---------+---------+---------+---------+---------+---------+---------|
Ref       ATGGATGCCAAGAAGTTTATGCAGTTGGTCGAGGAGAAAAAGAAGAGAGCTCTTGAGAAGAAAGAAGGCCACTTGAAATGGGAGCAAAAACTTGAAGCTTCAGCTAATTTGAAGTCTGATGCTGAAGCTG
RB        ATGGATGCCAAGAAGTTTATGCAGTTGGTCGAGGAGAAAAAGAAGAGAGCTCTTGAGAAGAAAGAAGGCCACTTGAAATGGGAGCAAAAACTTGAAGCTTCAGCTAATTTGAAGTCTGATGCTGAAGCTG
Consensus ATGGATGCCAAGAAGTTTATGCAGTTGGTCGAGGAGAAAAAGAAGAGAGCTCTTGAGAAGAAAGAAGGCCACTTGAAATGGGAGCAAAAACTTGAAGCTTCAGCTAATTTGAAGTCTGATGCTGAAGCTG

          131     140       150       160       170       180       190       200       210       220       230       240       250       260
          |--------+---------+---------+---------+---------+---------+---------+---------+---------+---------+---------+---------+---------|
Ref       GAGAGAAAACC---GCAGCTAAGCATAAAAGGAAATCTGACTCGGGGTCTGATAGTGGGAGTGACACTGACAGTGAATATGAAAAGAAAAAATCCACGAGAAAGTCCCATAAGAAGCACAGGAAGCACCA
RB        GAGAGAAAACCAAGGCAGCTAAGCATAGAAGGAAATCTGACTCGGGGTCTGATGATGGGAGTGACACTGACAGTGAATTTGAAAAGAAAAAATCCACGAGAAAGTCCCATAAGAAGCACAGGAAGCACCA
Consensus GAGAGAAAACC...GCAGCTAAGCATAaAAGGAAATCTGACTCGGGGTCTGATaaTGGGAGTGACACTGACAGTGAATaTGAAAAGAAAAAATCCACGAGAAAGTCCCATAAGAAGCACAGGAAGCACCA

          261     270       280       290       300       310       320       330       340       350       360       370       380       390
          |--------+---------+---------+---------+---------+---------+---------+---------+---------+---------+---------+---------+---------|
Ref       TAGCTCAAATTTGGTTGATTTGGATAGGAAGGACAAGAAATCCAAGCGGAAGCCAAAGAGAAGGTCCTCTGATTCCGGTGATGATAGCTGTAGGGAATATGATGGTGATTCAGAAGAAGAGAGAAGAAGA
RB        TAGCTCAGATTTGGTTGATTTGGATAGGAAGGACAAGAAAACCAAGCGGAAGCCAAAGAGAAGGTCCTCTGATTCCGGTGATGATAGCTGTAGGGAATATGATGGTGATTCAGAAGAAGAGAGAAGAAGA
Consensus TAGCTCAaATTTGGTTGATTTGGATAGGAAGGACAAGAAAaCCAAGCGGAAGCCAAAGAGAAGGTCCTCTGATTCCGGTGATGATAGCTGTAGGGAATATGATGGTGATTCAGAAGAAGAGAGAAGAAGA

          391     400       410       420       430       440       450       460       470       480       490       500       510       520
          |--------+---------+---------+---------+---------+---------+---------+---------+---------+---------+---------+---------+---------|
Ref       AAGAAGCAGGGCCACAAGAAGCGTAGGCATCATGATAGATACTCTGATTATAATTCCTCTGACTCTTCAGATGATGAAGACGTGGGAAGAAAACACCATTCAAAGCATCACAAACGTCATCGACGATCAC
RB        AAGAAGCGGGGCCACAAGAAGCATAGGCATCATGATAGATACTCTGATTATAATTCCTCCGACTCTTCAGATGATGAAGACGTGGGAAGAAAACACCATTCGAAGCATCACAAACGTCATCGACGATCAC
Consensus AAGAAGCaGGGCCACAAGAAGCaTAGGCATCATGATAGATACTCTGATTATAATTCCTCcGACTCTTCAGATGATGAAGACGTGGGAAGAAAACACCATTCaAAGCATCACAAACGTCATCGACGATCAC

          521     530       540       550       560       570       580       590       600       610       620       630       640       650
          |--------+---------+---------+---------+---------+---------+---------+---------+---------+---------+---------+---------+---------|
Ref       AACCGAATGGATCAGAGTCTTCCAGTGATGATGACCATGTTGCAGGTAGGAAACGAAGCAACGCAAAGCATCATAAACGTCATGGAAGGTTGGACTCAGATGCTCCTGTCTCTTCTAGCGATGAAGAAAA
RB        AACCGAATGGATCAGAGTCTTCCAGTGATGATGACCATGTTGCAGCTAGGAAACGAAGCAACACAAAGCATCATAAACGTCATGGAAGGTTGGACTCAGATGCTTCTGTCTATTCTAGCGATGAAGAAAA
Consensus AACCGAATGGATCAGAGTCTTCCAGTGATGATGACCATGTTGCAGcTAGGAAACGAAGCAACaCAAAGCATCATAAACGTCATGGAAGGTTGGACTCAGATGCTcCTGTCTaTTCTAGCGATGAAGAAAA

          651     660       670       680       690       700       710       720       730       740       750       760       770       780
          |--------+---------+---------+---------+---------+---------+---------+---------+---------+---------+---------+---------+---------|
Ref       AGTGAACCGTAGTAATCATGGAAAACATAAGAAACGTCATCACAGACCCCATAGTCACGACTCAAGGTCTTCGGATCCGGAAGATTTTAGGCATGAGAGAAGTAGATTCTTAGGTAAATCTTCTGATGAG
RB        AGTGAACCGTAGTAATCATGGAAAACATAAGAAACGTCATCACAGACACCATAGTCACGACTCAAGGTCTTCGGATCCTGAAGATTTTAGGCATGAGAGAAGTAGATTCTTAGGTAAATCTTCTGATGAG
Consensus AGTGAACCGTAGTAATCATGGAAAACATAAGAAACGTCATCACAGACaCCATAGTCACGACTCAAGGTCTTCGGATCCgGAAGATTTTAGGCATGAGAGAAGTAGATTCTTAGGTAAATCTTCTGATGAG

          781     790       800       810       820       830       840       850       860       870       880       890       900       910
          |--------+---------+---------+---------+---------+---------+---------+---------+---------+---------+---------+---------+---------|
Ref       AATGAGGAATTAGATAGAAAAGATAAGCACAGAAAGAATCATCATCGACATGGTCATCATCATCATCACCATTCCAACCATACGCATCGCCGCTCAGTTGAAGTGTCGAAGGACAACCATCAACCTCATG
RB        AATGAGGAATTAGATAGAAAAGATAAGCACAGAAAGAATCATCATCGACATGGTCGTCGTCGTCATCACCATTCCAACCATACGCATCGCCGCTCAGTTGAAGTGTCGAAGGACAACCATCAACCTCATG
Consensus AATGAGGAATTAGATAGAAAAGATAAGCACAGAAAGAATCATCATCGACATGGTCaTCaTCaTCATCACCATTCCAACCATACGCATCGCCGCTCAGTTGAAGTGTCGAAGGACAACCATCAACCTCATG

          911     920       930       940       950       960       970       980       990       1000 1005
          |--------+---------+---------+---------+---------+---------+---------+---------+---------+----|
Ref       ACAAAAAGGAAAGAAATGGCGAGCGAGTGGACCATACTTCTGAGATGCTCAAGGAAAGTGATAACAATGGTGCTCGTGTGATTCAAAATGCCTAG
RB        ACAAAAAGGAAAGAAATGGCGAGCAAGTGGACCATACTTCTGAGATGATCAAGGAAAGTGATAACAATGGTGCTCGTGTGATTCAAAATGCCTAG
Consensus ACAAAAAGGAAAGAAATGGCGAGCaAGTGGACCATACTTCTGAGATGaTCAAGGAAAGTGATAACAATGGTGCTCGTGTGATTCAAAATGCCTAG
```

d) CRISP-Cas9 based silencing of *StHRC* gene (*Sthrc*), showing deleted alleles

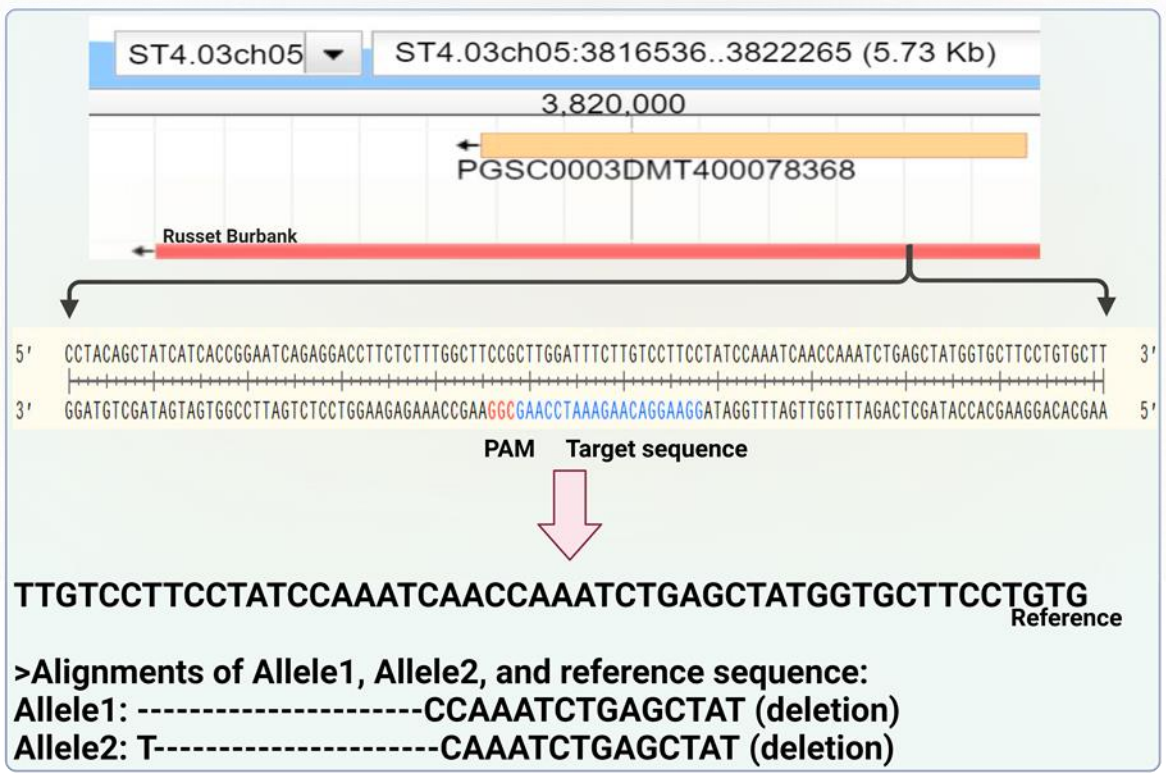

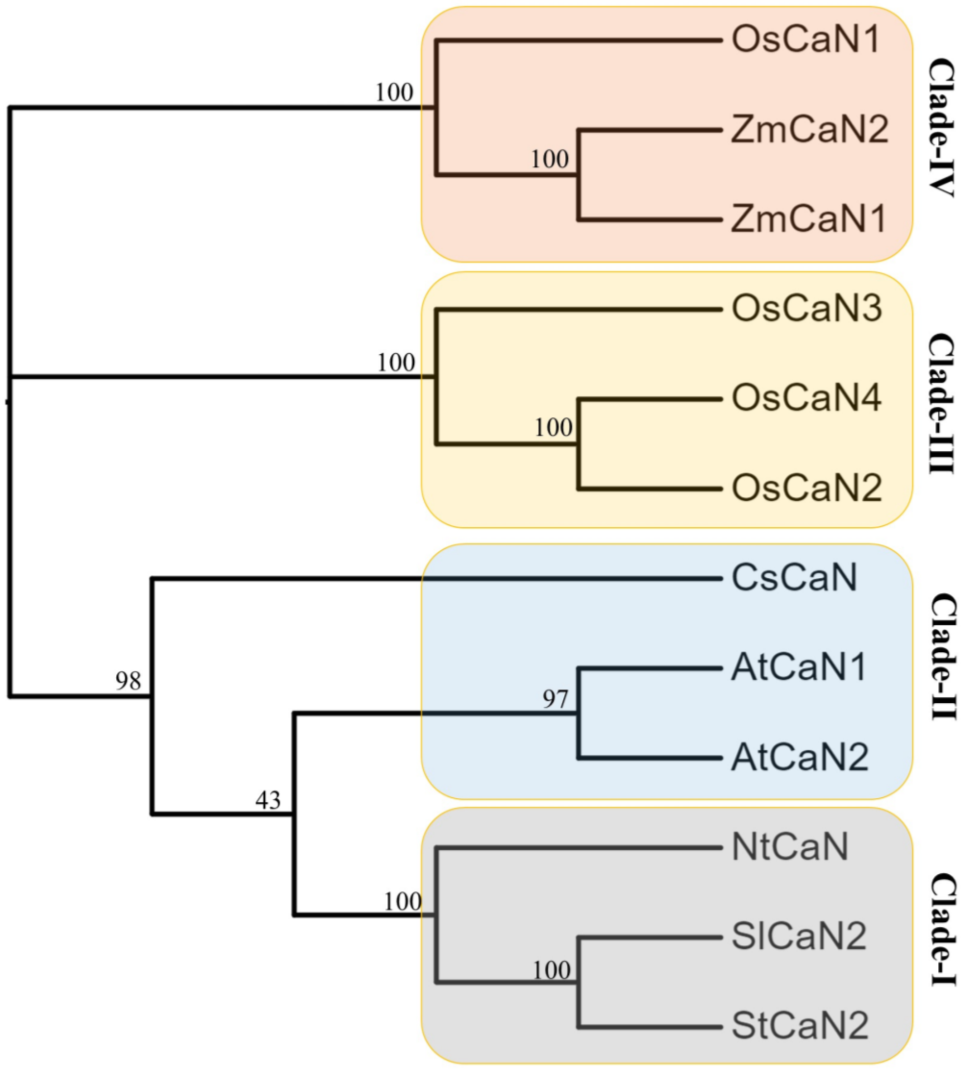

e) Phylogenetic tree of endonuclease gene *StCaN2* in selected plant species, showing four Clades, where the *StCAN2* belongs to Clade-I.

f) Amino acid sequence alignment of StCAN2 genes in plants. The putative staphylococcal nuclease (SNase) catalytic domain is shown in black box. Black circles indicate Ca2+ binding site. The open circles show the active site of the SNase catalytic domain.

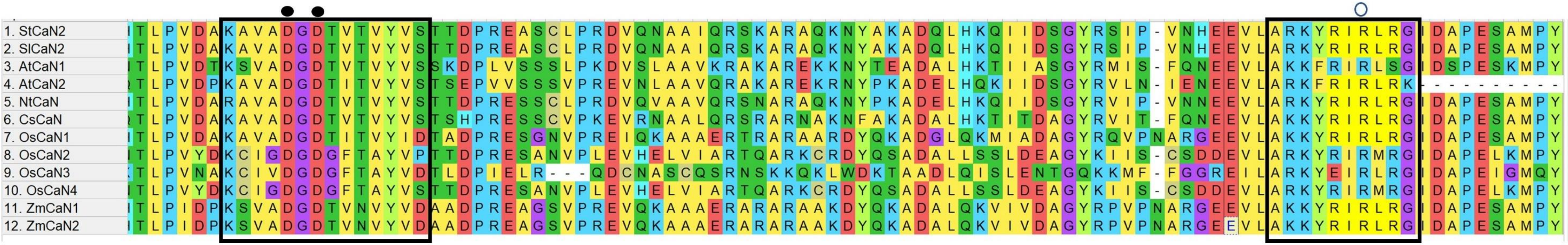

g) Agarose gel images for DNA laddering of StHRC/sthrc inoculated with mock and *P. infestans*, where: M1– 1Kb plus DNA marker, M2 – 100bp DNA marker. 1- *StHRC* mock; 2- *StHRC* pathogen; 3- *sthrc* mock; 4- *sthrc* pathogen inoculated.

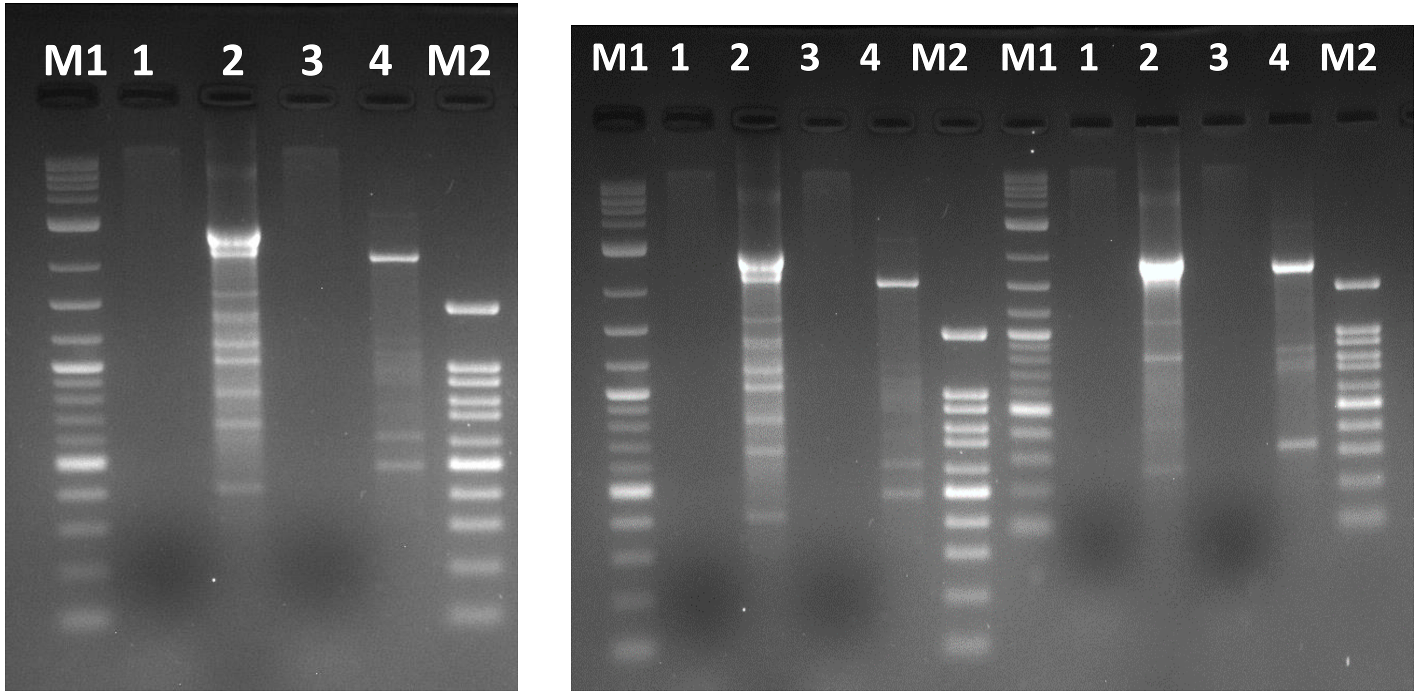

h) Agarose gel images for DNA laddering of StHRC/sthrc inoculated with mock and A. solani, where: M1– 1Kb plus DNA marker, M2 – 100bp DNA marker. 1- *StHRC* mock; 2- *StHRC* pathogen; 3- *sthrc* mock; 4- *sthrc* pathogen inoculated.

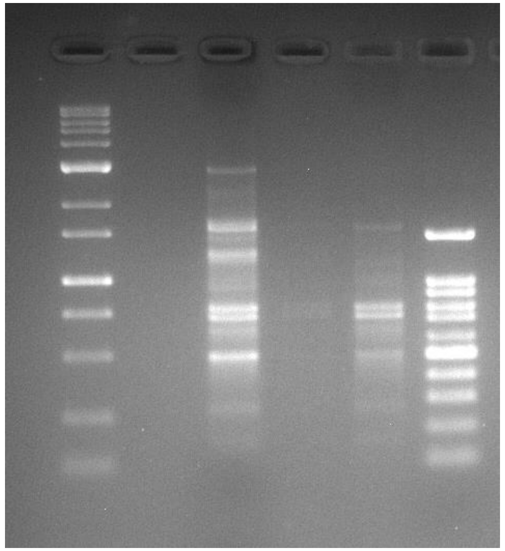

Supplement: Supplementary file 1 — Supplementary Figure S1. [file 41598_2022_24831_MOESM1_ESM.pdf]
