# Supplementary material for: Apoptotic-like PCD inducing HRC gene when silenced enhances multiple disease resistance in plants
Source: Sci Rep. 2022 Nov 27;12:20402. doi: 10.1038/s41598-022-24831-0 (PMC9701806; doi:10.1038/s41598-022-24831-0)
Supplement: Supplementary file 2 — Supplementary Table S1. [file 41598_2022_24831_MOESM2_ESM.pdf]

Apoptotic-like PCD inducing HRC gene when silenced enhances multiple disease resistance in plants

Kushalappa AC[1,5*], Hegde NG[1,5], Gunnaiah R[1,2,5], Sathe A[1], Yogendra KN[3], and Ajjamada L[4]

Scientific Reports: Supporting information

Table S1. The primers used in this study

**Genes**	**Gene ID**	**Primers**
TaHRC-wheat NILs (*TaCaMBP_Fhb1*)	*TAA_ctg0954b.00390.1*	F: 5'-TCAGGTTCTGAGGCATTTTAACAAC-3'<br>R: 5'-GAGGAACGACTTTTCGCTGGT-3'
StHRC sequencing primers		
StHRC- RB potato (900bp)	PGSC0003DMG400030513	F: 5'-AGCCTGAAGTCCTTCGTGAAT-3'<br>R: 5'-GGAGCATCTGAGTCCAACCT-3'
StHRC- RB potato (400bp)	PGSC0003DMG400030513	TTTATGCAGTTGGTCGAGGAGA<br>TACGCTTCTTGTGGCCCTG
**CRISPR Cloning sgRNA cloning in pDIRECT21A**		
StHRC_sgRNA		F:5'-GATTGGAAGGACAAGAATCCAAG-3'<br>R: 5'-AAACCTTGGATTCTTGTCCTTCC-3'
**Pathogen biomass quantification**		
O-8 (*Phytophthora infestans*)	GQ371195.1	F: 5'-GAAAGGCATAGAAGGTAGA-3'<br>R: 5'-TAACCGACCAAGTAGTAAA-3'
ITS (*Alternaria sp.*)		F: 5'-TCCGTA GCTGAACCTGCGG -3'<br>R: 5'-TGGGTTGGTCCTTGTGGTG-3'
txtAB (*Streptomyces scabies*)	AF255732 (*S. acidiscabies*)<br>AY707081 (*S. turgidiscabies*)	F: 5'-GCAGGACGCTCACCAGGTAGT-3'<br>R: 5'- ACTTCGACACCGTTGTCCTCAA-3'
Stb-tubulin	Z33382.1	F: 5'-ATGTTCAGGCGCAAGGCTT-3'<br>R: 5'-TCTGCAACCGGGTCATTCAT-3'
StEf1-α	AB061263.1	F: 5'- ATTGGAAACGGATATGCTCCA-3'<br>R:5'- TCCTTACCTGAACGCCTGTCA-3'
***qRT-PCR gene* expression**		
StHRC- RB potato	PGSC0003DMG400030513	F: 5'-AGATGCACTTCTGGTCATCATC-3'<br>R: 5'-CTCCTCGACCAACTGCATAAA-3'
StCaN2 – RB potato	PGSC0003DMG400017899	F: AGAAACAAAGCCTCCTCCTAAA<br>R: TGAGGAAGAGGAAGACCATAGA
StActin	PGSC0003DMG400023429	F: GCTTCCCGATGGTCAAGTCA<br>R: GGATTCCAGCTGCTTCCATTC
Stb-tubulin	Z33382.1	F: 5'-ATGTTCAGGCGCAAGGCTT-3'<br>R: 5'-TCTGCAACCGGGTCATTCAT-3'
StEf1-α	AB061263.1	F: 5'-ATTGGAAACGGATATGCTCCA-3'<br>R: 5'-TCCTTACCTGAACGCCTGTCA-3'
SotubMC7-F (StMC7)	PGSC0003DMG400011909	F: 5'-GCCTTCTCGAGTAGCTGTTGA-3'<br>R: 5'-TCACATGATGGAGAATGGTT-3'
